# Orthorexia Nervosa—It Is Time to Think About Abandoning the Concept of a Distinct Diagnosis

**DOI:** 10.3389/fpsyt.2021.640401

**Published:** 2021-01-28

**Authors:** Adrian Meule, Ulrich Voderholzer

**Affiliations:** ^1^Department of Psychiatry and Psychotherapy, University Hospital, Ludwig Maximilian University of Munich, Munich, Germany; ^2^Schoen Clinic Roseneck, Prien am Chiemsee, Germany; ^3^Department of Psychiatry and Psychotherapy, University Hospital of Freiburg, Freiburg, Germany

**Keywords:** orthorexia nervosa, anorexia nervosa, bulimia nervosa, eating disorders, obsessive-compulsive disorder

## Introduction

The term orthorexia nervosa (ON) refers to an obsessive focus on healthy eating (1). Proposed diagnostic criteria include a dietary theory or set of beliefs about healthy foods, exaggerated emotional distress in relationship to food choices that are perceived as unhealthy, and clinical impairment because of compulsive dietary behaviors and mental preoccupation (2). Although research on ON has been conducted for 20 years, its measurement and clinical value are still hotly debated. For example, it is not clear whether ON is actually a diagnostic entity that is distinct from established eating disorders and other mental disorders such as obsessive-compulsive disorder.

## Measurement of On

The large majority of studies on ON have been based on a questionnaire measure called the ORTO−15 (3) or several short versions of it [e.g., (4–6)]. However, studies have shown consistently that this instrument has poor psychometric properties, the most prominent of which is its low internal reliability [e.g., (7–9)]. Thus, findings based on the ORTO−15 are inconsistent and hardly interpretable. Several other instruments have been developed [cf. (9)]. However, using these measures has not been recommended [e.g., Bratman's Orthorexia Test (1); cf. (8, 9)] or they have only been used in a handful of studies yet [e.g., the Eating Habits Questionnaire (10) or the Orthorexia Nervosa Inventory (11); cf. (9, 12)]. A relatively new measure with sound psychometric properties and which is increasingly used internationally (13–16) is the Düsseldorf Orthorexia Scale [DOS; (17)]. Therefore, we focus on studies that used the DOS in this opinion piece.

## Overlaps and Differences Between ON and Eating Disorders

A key feature of ON is that food choices are based on qualitative, health-related aspects of foods. That is, unlike in persons with anorexia nervosa, food choices are not based on quantitative, energy density-related aspects of foods that are motivated by a drive for thinness and body dissatisfaction. Specifically, Cena et al. (12) argue that—while established eating disorders and ON share common characteristics such as a concern over food and eating—ON is marked by open, rationalized rules related to eating and a focus on the quality of foods instead of fears of gaining weight and body image disturbances. Unlike eating disorders, which are more prevalent in females than males, conceptualizations of ON do also assume that there is no sex difference in orthorexic symptomatology (12). However, most studies that used the DOS could not demonstrate that orthorexic symptoms are independent from features of disordered eating behavior. For example, positive correlations between DOS scores and eating disorder symptoms such as drive for thinness and body dissatisfaction have been found in both non-clinical and clinical samples [e.g., (18–20)] and orthorexic symptomatology tends to be higher in females than males [e.g., (21)].

In line with these reports, we found that prevalence rates of ON (based on DOS scores of ≥30) were high in inpatients with anorexia nervosa (48%) and bulimia nervosa (33%) in a recent study that tested a large sample of inpatients with mental disorders (22). Furthermore, DOS scores significantly decreased from admission to discharge, although orthorexic eating was not targeted in the eating disorder-specific treatment. Finally, higher DOS scores strongly related to higher drive for thinness and higher body dissatisfaction in these groups (*r* ≥ 0.5). Thus, our findings speak against the notion that ON represents a distinct diagnostic entity and suggest that it may rather be an aspect of restrictive eating disorder symptomatology.

## Overlaps and Differences Between On and Obsessive-Compulsive Disorder

Cena et al. (12) not only discuss similarities and differences between ON and eating disorders but also between ON and obsessive-compulsive disorder. Specifically, they highlight rigidity, perfectionism, and other obsessive-compulsive features as common characteristics while the only difference being that obsessive-compulsive symptoms relate to food and eating in ON. Similarly, other authors have described the rigid beliefs about healthy eating as a form of obsessive thoughts and the avoidance of subjectively perceived unhealthy foods as a form of compulsive behavior [e.g., (23)] and higher DOS scores indeed relate to higher obsessive-compulsive symptoms [e.g., (24)].

In our study in inpatients with mental disorders (22), however, we did not find a substantial overlap between ON and mental disorders other than eating disorders. That is, the prevalence of ON (based on DOS scores of ≥30) was similar to prevalence rates reported in the general population [0–3%; e.g., (17, 20, 21)] in groups of patients with a depressive episode, recurrent depressive disorder, phobic disorders, obsessive-compulsive disorder, trauma-related disorders, and somatoform disorders. Unlike the changes observed in patients with eating disorders, DOS scores did not change from admission to discharge in these groups. Thus, this study showed a clear distinction from other mental disorders such as obsessive-compulsive disorder, which is in line with other reports (25).

## Discussion

We conclude that—although orthorexic symptoms seem to be distinct from mental disorders such as obsessive-compulsive disorder—this distinction does not hold for established eating disorder diagnoses. Although features of eating disorders such as drive for thinness, body dissatisfaction, and sex differences are not part of ON conceptually, the independence of orthorexic symptomatology from these features could not be demonstrated empirically. To put our results into perspective, however, we also need to mention that DOS scores were unrelated to features of disordered eating in two studies from China (15, 26), indicating that there might be cultural differences in orthorexic symptomatology or in its measurement. Cultural differences—between China and Western countries in particular—have been previously noted in self-report measures of eating behavior and their correlates with the reasons for these differences remaining elusive (27, 28). Thus, we cannot exclude that the current evaluation about the ON construct is limited to Western (particularly European) countries, in which the majority of studies were conducted.

As a final remark, we argue that future research on ON requires more sophisticated approaches than cross-sectional questionnaire studies. For example, large interview-based studies seem to be necessary to gain deeper insights into the ON construct. Such studies may include applying structured clinical interviews such as the SCID−5–RV (29) and additionally the DOS or, preferably, a newly developed ON-specific interview. Only when individuals show an orthorexic eating behavior (e.g., as indicated by DOS scores ≥30) that is accompanied by a clinical impairment or subjective distress *and* at the same time do not receive an established eating disorder diagnosis (which not only includes anorexia nervosa and bulimia nervosa but also any of the other eating disorder diagnoses listed in the DSM−5 or ICD−11), then a distinct diagnosis of ON may be justified. As results from empirical studies indicate, however, we would expect that such cases are rarely found (or may not be found at all) in the general population.

## Author Contributions

UV conceived the topic and proposition of this manuscript. AM wrote the first draft. Both authors contributed to revision of the manuscript.

## Conflict of Interest

The authors declare that the research was conducted in the absence of any commercial or financial relationships that could be construed as a potential conflict of interest.
